# The Book World of Medicine and Science

**Published:** 1900-02-03

**Authors:** 


### The Book World of Medicine and Science.

A System of Medicine by Many Writers. Edited by Thomas Clifford Allbutt, M.A., M.D., F.R.C.P., F.R.S. (Volume VIII. London: Macmillan and Co. 1899. Price 25s.)

With this the eighth and final volume of the series, "Allbutt's System of Medicine" is completed, and a work is finished which will for a long time to come remain the authoritative exposition of the position of medicine at the end of the nineteenth century. The feature of this work which most strongly strikes us as we turn over the pages, not of one volume alone, but of the whole series as they now lie before us, is the widely representative character of the contributions by which they are filled. Everywhere we find that its pages contain records and descriptions given at first hand by actual workers in the various subjects treated of, and while the Editor has evidently taken care that each chapter shall offer a general survey of its own topic, the fact that this survey is in most cases given by men who are themselves workers in that special field removes the book entirely from the category of the compilations. The title "A System of Medicine" is the old one with which the elder among us have long been familiar, but from the end of the title to the end of the book all the rest is original and absolutely modern.

Turning now more particularly to this final volume we find that it contains the completion of diseases of the nervous system, this part occupying about 170 pages; an admirable account of mental diseases by various authors, "edited with the kind help of Dr. Savage," occupying about 300 pages; and then a very full and complete section on diseases of the skin, also by various authors, and "edited with the kind help of Dr. Payne." Both the latter sections contain a very large amount of practical information, and may almost be regarded as complete works on their respective subjects. The volume commences with a very readable and interesting article on craft palsies by Dr. Vivian Poore. Then come articles on various other forms of spasm, on migraine by Dr. James Mackenzie, on paralysis agitans by Sir W. Gowers, on hysteria by Dr. Ormerod, on neurasthenia by the Editor, and on traumatic neurasthenia by Mr. Horsley. This finishes the section on diseases of the nervous system. In this group of articles the two on neurasthenia are certainly the most striking, and should be read by all practitioners, for it is to be feared that, besides occurring as a fully marked and recognisable disease, this neurasthenia, or, at any rate, a neurasthenic condition, which at root is the same thing, forms an important complication of the sufferings of many a man whose friends—and, maybe, even his doctor—are "sure that a great deal of his complaint is fancy," readily as they may admit he is not well. The neurasthenic side of the common ailments of daily life is a matter well worthy of the study of general practitioners.

In regard to the management of this disease both Professor Allbutt and Professor Horsley agree as to the great efficacy of the Weir-Mitchell, or, as the former calls it, the Mitchell-Playfair method of treatment. Horsley says, "A wide experience of traumatic neurasthenia shows that no case can be expected to end in recovery if the symptoms be severely marked unless treated by the Weir-Mitchell method of counteracting ordinary idiopathic neurasthenia." Allbutt, however, in speaking of the idiopathic form, is by no means so exclusive. Perhaps he is also less hopeful; but it must be borne in mind that so far as the non-traumatic neurasthenia is concerned the condition in some persons "seems rather to be the expression of a permanent and hereditary quality than an intercurrent disease," and Allbutt says very truly that "the spare, weary, aching patients who are fit for the Weir-Mitchell method may receive a more confident prognosis of amendment than the robuster and more active-bodied patients whose symptoms are more exclusively cerebral." The position of these articles in the volume seems not inappropriately chosen, for they are placed within easy reach of disorders of the mind. In the section on mental diseases there is much that will be found useful and illuminating, and some of the articles are of very great value. It is to be noted that, owing probably to the division of the subjects among the several authors, some of the mental disorders treated of are regarded from two separate points of view—that of the causation and that of the state produced. Thus, while there are separate chapters on "The Epochal Insanities," "Toxic Insanities," "Alcoholic Insanity," &c., there are also separate chapters treating of the several forms which mental disturbance, however caused, may assume, such as Mania, Acute Delirium, Melancholia, Stupor, Recurrent Insanity, &c. Among the many very excellent articles in this section it would be invidious to select any for especial praise, but as giving food for thought, and as being somewhat out of the common run, attention may well be drawn to the article by Dr. Mercier on "Vice, Crime, and Insanity." It is a picturesquely-worded article, playing upon the idea of self-control, and tending to drive home the view that the lack of self-control, which separates man from brute, and civilised man from savage, is also the key to the contrast between morality and immorality, between vice and chastity, and between sanity and insanity. The description given of the mode in which obsession may lead to crime, and the parallelism drawn between such obsession and acts which are within the experience if not of all, at least of many of us, is very striking, although perhaps the picture drawn of a divided self, one part warring against another, may not be acceptable to all. An article which will be found very useful by practitioners, and especially by those whose experience in matters of lunacy is not great, is that on "English Law and Practice of Lunacy," by Dr. Outterson Wood and Dr. Savage.

The section dealing with "Diseases of the Skin" occupies nearly 500 pages, and will be found to be very practical and complete. It is full of information, not merely in regard to the more ordinary diseases, but about a host of strange morbid conditions which no doubt many of us have never met with. When we add that among the contributors to this section there appear the names of Dr. Payne, Dr. Stephen Mackenzie, Mr. Jonathan Hutchinson, Dr. Radcliffe Crocker, Dr. Head, Dr. Pringle, Dr. Phineas Abraham, Dr. Galloway, Dr. Colcott Fox, Dr. Manson, and Mr. Anderson, and that, besides contributing other articles, Mr. Malcolm Morris takes charge of the chapter on the vegetable parasitic diseases of the skin, it will be recognised at once with what authority the book speaks on all matters pertaining to this department of medicine. In an appendix, Dr. Patrick Manson details the present position of our knowledge of malaria, the recent discoveries in the life history of the malaria parasite, which have been made since the appearance of Dr. Osler's article in Vol. II., rendering the insertion of such an appendix, as the editor says, "more than justifiable." This volume forms a worthy conclusion to a most admirable work.

